# Proteome changes of plasma-derived extracellular vesicles in patients with myelodysplastic syndrome

**DOI:** 10.1371/journal.pone.0262484

**Published:** 2022-01-10

**Authors:** Klara Pecankova, Pavla Pecherkova, Zdenka Gasova, Zofie Sovova, Tomas Riedel, Eliézer Jäger, Jaroslav Cermak, Pavel Majek

**Affiliations:** 1 Institute of Hematology and Blood Transfusion, Prague, Czech Republic; 2 Institute of Macromolecular Chemistry CAS, Prague, Czech Republic; East China Normal University School of Life Sciences, CHINA

## Abstract

**Background:**

Extracellular vesicles are released into body fluids from the majority of, if not all, cell types. Because their secretion and specific cargo (e.g., proteins) varies according to pathology, extracellular vesicles may prove a rich source of biomarkers. However, their biological and pathophysiological functions are poorly understood in hematological malignancies.

**Objective:**

Here, we investigated proteome changes in the exosome-rich fraction of the plasma of myelodysplastic syndrome patients and healthy donors.

**Methods:**

Exosome-rich fraction of the plasma was isolated using ExoQuick^™^: proteomes were compared and statistically processed; proteins were identified by nanoLC-MS/MS and verified using the ExoCarta and QuickGO databases. Mann-Whitney and Spearman analyses were used to statistically analyze the data. 2D western blot was used to monitor clusterin proteoforms.

**Results:**

Statistical analyses of the data highlighted clusterin alterations as the most significant. 2D western blot showed that the clusterin changes were caused by posttranslational modifications. Moreover, there was a notable increase in the clusterin proteoform in the exosome-rich fraction of plasma of patients with more severe myelodysplastic syndrome; this corresponded with a simultaneous decrease in their plasma.

**Conclusions:**

This specific clusterin proteoform seems to be a promising biomarker for myelodysplastic syndrome progression.

## Introduction

Myelodysplastic syndrome (MDS) encompasses a diverse range of oncohematological diseases that affect hematopoietic stem cells and their microenvironment. It is characterized by ineffective hematopoiesis, blood cytopenias, and progression to acute myeloid leukemia. According to the WHO classification [1], which is based on cytogenetics and findings in the bone marrow and peripheral blood, MDS patients are divided into several subgroups. Refractory anemia (RA) and refractory anemia with ringed sideroblasts (RARS) are MDS subgroups characterized by dysplasia limited to erythroid lineage, by less than 5% of the blasts being located in the bone marrow, and by limited response to treatment. Another subgroup is refractory cytopenia with multilineage dysplasia (RCMD), which is defined by the presence of varying degrees of peripheral blood cytopenia and by dysplastic changes that are present in 10% or more of the cells in two or more myeloid lineages in the bone marrow (with less than 15% ringed sideroblasts). Refractory anemia with excess of blasts type one and two (RAEB-1, RAEB-2) are other subgroups; they are recognized on the basis of the number of these blasts in the bone marrow: RAEB-1 with 5–9% and RAEB-2 with 10–19% [2].

Extracellular vesicles (EVs) are small membrane vesicles released into body fluids from the majority of, if not all, cell types and cancer cells [3]. They are present in plasma, urine, breast milk, semen, amniotic fluid, ascites, saliva, interstitial fluid, and extracellular matrix [4–13]. EVs contain and transport a wide range of cargo, including mRNA, miRNA, proteins (membrane and cytosolic), enzymes, transcription factors, molecular chaperones and signaling molecules. Differences in their cargo may reflect their function [14–18]. EV functions are thought to include intercellular communication [19, 20], immune surveillance [21], stem cell maintenance [22], tissue regeneration [23] and blood coagulation [24].

EV secretion is known to be dependent on pathology (e.g., cancer, inflammation, hematological disorders). For example, hypoxia or oxidative stress can trigger EV secretion [25]. While the involvement of EVs in hematological malignancies has been poorly investigated [18, 25], various cancer cell lines have been shown to secrete more EVs than normal cells. In B-cell chronic lymphocytic leukemia and in mouse multiple myeloma, the total EV level was significantly higher than in healthy subjects [26, 27]. More importantly, the EV cargo in cancer cells is distinct from that in healthy ones and is highly variable according to cell origin [28].

In recent years, MS-based proteomics has been used to identify vesicular proteins and have helped reveal the protein composition of EVs from various cell types and body fluids [29], including plasma [30, 31]. Several distinct features make EVs an attractive source for proteomic research. They are rich in low-abundance proteins that are underrepresented in unfractionated biological materials. They contain a specific set of proteins that can help determine the environment from which the EVs originated. Therefore, they could prove to be a rich source of biomarkers [18, 32].

The results of several proteomic studies have provided insight into how exosomes contribute to the pathobiology of hematological malignancies. For example, it was suggested that in multiple myeloma the proteins transferred by exosomes to malignant cells can promote tumor growth and spreading [33]. Proteins identified in exosomes derived from human lymphoma cells were associated with either antigen presentation and processing or cell migration, suggesting that exosomes play an important role in immunity regulation and the interaction between lymphoma cells and their microenvironment [34]. Paggetti *et al*. [35] showed that chronic lymphocytic leukemia-derived exosomes actively promote disease progression by modulating the surrounding stromal cells. The proteomic profiling of AML serum exosomes revealed both up- and downregulated protein patterns compared with exosomes isolated from the sera of healthy donors [36].

However, to date, these promising studies are the exception rather than the rule. More studies on the protein and other molecular content of exosomes are needed to decipher their potential function in hematological malignancies. Thus, we here investigate and report on proteome changes in the exosome-rich fraction of the plasma (ERF) of MDS patients.

## Materials and methods

### Sample collection

We analyzed 36 MDS patient samples (RA and RARS n = 12, RCMD n = 12, RAEB-1 and RAEB-2 n = 12) and 12 healthy donor control samples. Patient diagnosis of MDS was established according to the WHO classification criteria [37]. The median age of the MDS patients was 65 and the group included 18 females (50%). The median age of the gender-matched control subjects was 41. Patients were selected on the basis of their diagnosis and those who agreed to participate in the study provided written informed consent. All samples were obtained and analyzed in accordance with the Helsinki Declaration and the regulations of the Ethical Committee of the Institute of Hematology and Blood Transfusion. The study was approved by the Ethical Committee of the Institute of Hematology and Blood Transfusion.

Venipuncture was used to draw the blood samples into EDTA-coated tubes. Plasma was obtained by centrifugation at 4,000 × g for 5 min, aliquoted and stored at -70°C until used. Prior to ERF isolation, the thawed plasma samples were centrifuged at 17,500 × g for 5 min and defibrinated using thrombin (500 UI/ml, Sigma Aldrich, Czech Republic) according to the manufacturer’s instructions for ExoQuick^™^ precipitation reagent (System Biosciences, Palo Alto, CA, USA).

### ERF isolation

The ERF was isolated from the defibrinated plasma using a modified protocol for ExoQuick^™^ precipitation reagent used by Hrustincova *et al*. [38]. To obtain fractions with low levels of plasma protein contamination, the manufacturer’s protocol was modified by the incorporation of several additional washing steps involving ExoQuick^™^. Defibrinated plasma (167 μl) with the addition of 1% Halt^™^ protease and phosphatase inhibitor cocktail (Thermo Scientific, Rockford, IL, USA) was mixed with ExoQuick^™^ (42 μl), incubated for 30 min at 4°C and then centrifuged at 1,500 × g for 30 min. After discarding the supernatant, the pellet was resuspended in PBS (167 μl) and shaken for 30 min. After this, ExoQuick^™^ (42 μl) was added and the mixture incubated for 30 min at 4°C before being centrifuged at 1,500 × g for 30 min. This step was repeated three times. After the last centrifugation, the pellet was resuspended in PBS (200 μl). The enriched fraction was precipitated with the addition of four volumes of acetone, incubated for 60 min at -20°C and centrifuged at 15,000 × g for 10 min. The isolated pellets were stored at -70°C until used.

### Dynamic Light Scattering (DLS)

The DLS measurements were performed by using the Zetasizer NanoZS, Model ZEN3600 (Malvern Instruments, UK) equipped with a 633-nm He-Ne laser and operating at an angle of 173°. The software used to collect and analyze the data was the Dispersion Technology Software version 6.01 from Malvern. 1 mL of the Exosomes solution (diluted 10 times) was measured in single-use polystyrene half-micro cuvettes (Fisher Emergo, Landsmeer, The Netherlands) with a pathlength of 10 mm. The measurements were made at a position of 4.65 mm from the cuvette wall with an automatic attenuator and at a controlled temperature of 25°C. For each sample, 1 run of 45 s were performed, with 10 repetitions. The size distribution and the polydispersity index (PDI) were obtained from the autocorrelation function using the “general purpose mode”.

### Protein electrophoresis

The protein concentrations of all samples were determined using Pierce^®^ 660 nm Protein Assay Reagent (Thermo Scientific, Rockford, IL, USA). The protein sample concentrations were then adjusted to the same level.

Isoelectric focusing, performed as previously described in detail [39] (IPG strips pI 4–7, 7.7cm; 50 μg of proteins/IPG strip), was followed by SDS-PAGE (8 × 10cm, 10% resolving gel, 3.75% stacking gel, 200 V constant voltage). The gels were silver stained according to Chevallet’s protocol [40] before being digitized (1200 dpi, 16-bit grayscale) and processed by Progenesis SameSpots software (Nonlinear Dynamics, Newcastle upon Tyne, UK), which computed the fold and *P* values of all spots using one-way ANOVA analysis. For the purpose of protein identification, a 2D preparative gel stained by colloidal Coomassie Blue was prepared.

### Mass spectrometry

Protein spots that differed significantly (*p* < 0.05) were submitted for protein identification by a tandem mass spectrometer (HCT ultra ion-trap mass spectrometer with nanoelectrospray ionization; Bruker Daltonics, Bremen, Germany) coupled with an RSLCnano system UltiMate 3000 (Thermo Scientific, Rockford, IL, USA). Tryptic peptides were desalted on a 300 μm ID/5 mm length C18 PepMap 100 precolumn (Dionex, Sunnyvale, CA, USA) and separated on a 75 μm ID/15 cm length C18 PepMap 100 analytical column (Dionex). A gradient of acetonitrile was used to elute the peptides (0% to 5% B in 1 min; 5% to 35% B in 30 min; 35% to 85% in 1 min; 85% for 5 min; 85% to 0% in 2 min; 0% for 18 min; mobile phase A—2% ACN/0.1% formic acid; mobile phase B—ACN/0.1% formic acid) at a flow rate of 300 nL/min. Standard-Enhanced positive scan mode was used for MS/MS; the scan ranges were 300–1500 m/z and 100–2500 m/z for MS and MS/MS, respectively. Three precursor ions were selected during one autoMS/MS cycle and active exclusion (3 spectra, 1 min; singly charged ions) was used. HyStar v3.2 and esquireControl v6.2 software were used for data acquisition and DataAnalysis v4.2 was used for data processing (all Bruker Daltonics). For database searching, BioTools v3.2 (Bruker Daltonics) combined with Mascot v2.2 (Matrix Science, London, UK) was used (UniProt human reviewed proteome [41]) with the following parameters: carbamidomethyl (C) as a fixed modification; oxidation (M) as a variable modification; number of missed cleavages up to 1 (up to 5 for error-tolerant search); monoisotopic mass; mass tolerance of 0.1% for MS and 1 Da for MS/MS for protein identification. Both CID (collision-induced dissociation) and ETD (electron transfer dissociation) fragmentations were used, after which Mascot was again used for database searching (Swiss-Prot). Two unique peptides (with higher Mascot scores than the minimum for identification, *p* < 0.05) were necessary to successfully identify a protein. To search for possible posttranslational modifications (PTMs) of the identified proteins, error-tolerant search (ETS) was performed on both CID and ETD data.

### Data analysis

The list of identified proteins was compared with ExoCarta, the exosome protein database [42–45], and with the list of exosomal proteins provided by QuickGO (EMBL-EBI) [46]. Comparisons were made by organism (*Homo sapiens*), UniProt accession number (AC) and/or by UniProt gene symbol.

### Statistical analysis

Progenesis SameSpots software was used to compare proteomes (RA and RARS n = 12, RCMD n = 12, RAEB-1 and RAEB-2 n = 12, and control samples n = 12) using ANOVA with the following minimum criteria *p* < 0.05 and fold ≥ 1.5. With respect to the two independent unequal-sized groups (MDS patients n = 36, healthy donors n = 12), the Mann-Whitney U test was used to differentiate between the groups. For both cohorts (MDS patients n = 36, healthy donors n = 12), correlations among spots were computed by the Spearman test using pairwise comparison. For the selected spot groups, their normalized volumes were correlated and the calculated correlation coefficients were compared between the MDS and the control group. The Spearman correlation coefficient has been used for all pairs of the variables. For all statistical analyses, the number of samples corresponded to the number of patients and controls (MDS n = 36, healthy controls n = 12). Level of significance was 0.05. All *P* values were interpreted descriptively, and no adjustment of *P* values or significance levels was applied. Statistical analysis was performed using IBM Statistical Package for the Social Sciences (SPSS) version 23 (IBM Corp. Armonk, NY, USA) and MATLAB R2021a (MathWorks Inc. Natick, MA, USA) software.

### ELISA

Clusterin levels in both the ERF and plasma were measured using commercial ELISA kit (ab174447, Abcam, Cambridge, UK). Samples were measured according to the manufacturer’s instructions.

### Deglycosylation assay

A deglycosylation assay was performed in accordance with Zielinska *et al*. [47] using peptide-*N*-glycosidase F (G1549, Sigma Aldrich, St. Louis, Missouri).

### Western blot

To study clusterin *N*-glycosylation patterns, as well as to monitor clusterin proteoforms in the ERF and blood plasma, 2D western blot analysis was performed. Pooled samples of both the ERF and plasma were prepared for each subgroup. All samples were normalized to the same protein amount. Following SDS-PAGE (10% resolving gel), the proteins were transferred to a PVDF membrane (10V constant voltage for 60 min) using an Owl HEP-1 semi-dry electroblotting system (Thermo Scientific, Waltham, MA, USA). After incubation with a blocking buffer (3% BSA in PBS) at 30°C for 60 min, the membranes were first incubated with primary antibody (CL7757 AP; Cedarlane, Burlington, Ontario, Canada) (1:10,000 dilution) and, then, with secondary goat anti-rabbit IgG (whole molecule) antibody conjugated with peroxidase (1:5,000 dilution) (A6154; Sigma-Aldrich, Prague, Czech Republic). Visualization was performed using a chemiluminescent substrate (SuperSignal West Pico; Thermo Scientific, Waltham, MA, USA) and CL-XPosure film (Thermo Scientific, Waltham, MA, USA). The 2D western blots were digitized (1200 dpi, 16-bit grayscale) and processed by Progenesis SameSpots software (Nonlinear Dynamics, Newcastle upon Tyne, UK). The expression profiles were based on spot normalized volumes.

## Results and discussion

**Fig 1:**
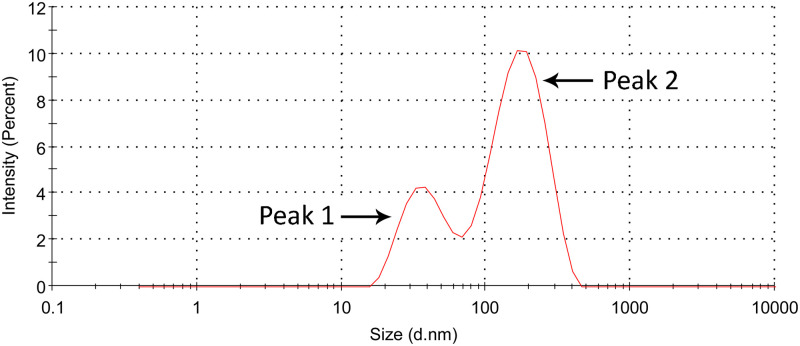
Characterization of the isolated ERF by DLS. DLS data interpretation was performed considering the parameter of intensity.

Although EVs isolated from blood plasma are a mixture produced by a variety of cells, most come from platelets and others from red and white blood cells [48–51]. EVs of other origin may indicate an oncological disease [52]. In view of the cell type distribution differences in MDS subgroups [48], we decided to isolate a complete set of plasma exosomes (ERF). By altering the manufacturer’s protocol for ExoQuick^™^, a fraction with a low level of plasma protein contamination (below 10%) was obtained. The identical protocol for EV isolation was used by Hrustincova *et al*. [38] who confirmed the presence and size of EVs by transmission electron microscopy, nanoparticle tracking analysis, and Western blotting (CD9 and CD81). To verify particle size distribution of isolated ERF in this work we used the DLS as a complementary technique. DLS showed the particle size distribution of the isolated fraction, with the majority of particles corresponding to exosome size. As shown in Fig 1, the particles exhibited a hydrodynamic size of 132 ± 22 nm with a relatively large polydispersity index (PDI) of 0.39 ± 0.14 which corresponded to an average value from 10 repeated measurements. The DLS data showed a multimodal distribution indicating particle size heterogeneity with two major contributions, consistent with previously published DLS data [53, 54]. According to the intensity parameter, particles with an average diameter of 39 nm (± 7 nm) accounted for about ~ 28% of the particle population (Fig 1, Peak 1), while larger particles that scatter light with greater intensity (150 nm ± 30 diameter) accounted for ~ 72% (Fig 1, Peak 2). An illustrative report measurement corresponding to one of the 10 repetitions, is also given as supplementary material (S1 File).

**Fig 2:**
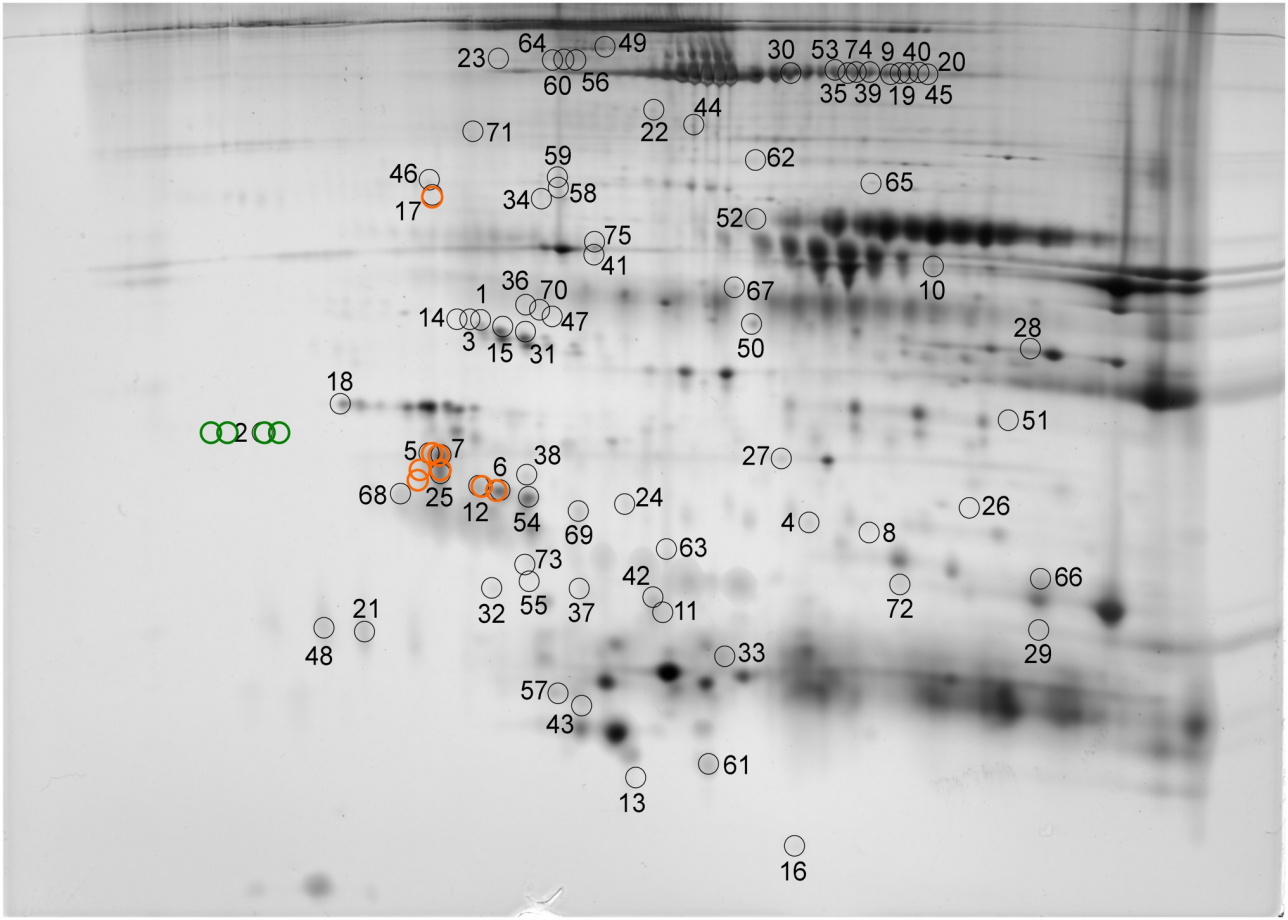
Positions of ERF proteome spots. Positions of ERF proteome spots significantly differing in normalized volumes (circles with numbers) and positions of spots selected on the basis of the Spearman correlation coefficient test (spots with normalized volumes correlating strongly in the MDS groups are highlighted in green; spots with normalized volumes correlating strongly in the control group are highlighted in orange). Brightness and contrast of the gel image were adjusted for clearer illustration.

By comparison of the ERF proteomes (RA-RARS, RCMD, RAEB and healthy volunteer control group), we found 75 spots that differed significantly (*p* < 0.05, ANOVA) in normalized volume (Fig 2—numbered circles). Analysis of the detected spots using nanoLC-MS/MS with CID and ETD identified 51 unique proteins, 90% of which matched exosome proteins in the ExoCarta, and QuickGO databases (S1 Table). S2 Table presents a list of all spots, including ANOVA *P* values, multiplication (fold value), protein identification with the number of identified peptides (unique peptides above the identity threshold score), and protein accession number (Swiss-Prot). The identified proteins belonged to various groups: acute phase proteins (e.g., alpha-1-antitrypsin, haptoglobin, serum amyloid P-component, fibrinogen); complement proteins (e.g., complement C3, C4, C5, C7, C1r subcomponent, mannose-binding protein C) and their regulators (clusterin, complement factor I, complement factor H, C4b binding protein); immunoglobulins; apolipoproteins; miscellaneous. It may seem surprising that immunoglobins were identified, but their presence could have been caused by plasma protein contamination, by exosomes released by B-cells and/or by the association of immunoglobulins with exosomal proteins [55, 56]. The same applies to apolipoproteins, which are structural elements of lipoprotein particles that have a similar size to that of exosomes and, thus, are often co-isolated. Currently, no technique is able to isolate exosomes from biological fluids in sufficient yield without protein or lipoprotein particle contamination [57–59]. Taking into account the participation of exosomes in lipoprotein metabolism [60], it is possible that lipoproteins are transported via exosomes to the point of destination. While apolipoproteins comprised just 10% of the identified proteins, it is possible that they contaminated exosomes (ERF) during isolation or that they resulted from the interaction of lipoprotein particles and isolated exosomes (ERF) [57].

The Mann-Whitney U test revealed 30 spots that distinguished (p = 0.001) the patient groups from the control group. Two groups of spots were selected based on the Spearman correlation coefficient test (*p* < 0.001). The first group of spots (spots 5, 6, 7, 12, 17 and 25) correlated strongly (0.8 < ρ^2^ < 1.0) in the control group (Fig 2—highlighted in orange) while the second (spot 2 and surrounding spots) correlated strongly in the MDS patients (Fig 2—highlighted in green). In the second group of spots, C4b binding protein was identified. Based on spot and unique peptide count, we decided to further analyze the first group of spots, which were mainly represented by clusterin, previously identified in human plasma exosomes [30].

Complement proteins participate in fighting pathogens and in removing dead or modified self cells. Their action on the surface of healthy self cells is inhibited by regulators in the blood plasma (clusterin, C4b binding protein) or by regulators bound to the cell surface [61]. Clusterin regulates the complement pathway at the point of terminal complement (membrane attack) complex formation. When the complement is inactivated, clusterin interacts with C7 (spot 65), C8 beta chain and C9 to prevent terminal complex formation [62, 63]. If regulatory molecules are altered, they do not perform their correct function. This can cause the opsonization of healthy self cell surfaces and lead to inflammation, autoimmunity or pathological states [61]. Alterations in clusterin have been observed in various cancer types, including renal, breast, ovarian, pancreatic or prostate cancer [64, 65].

Our 2D SDS-PAGE results cannot address the origin of clusterin changes because both protein expression alterations and PTMs are reflected in the proteome map. To determine whether clusterin is posttranslationally modified or differently expressed (or both), we used ELISA to estimate clusterin levels in both the isolated ERF and plasma. Plasma levels were measured to exclude potential interference with clusterin levels in ERF. In both the ERF and plasma, the clusterin levels were not related to MDS progression. This means that any clusterin change was caused by PTM(s).

Because approximately 30% of clusterin mass is composed of *N*-linked carbohydrates [62], we first focused on clusterin glycosylation. Glycosylation affects the clusterin chaperone function; complete deglycosylation corresponds to minimal chaperone activity [66]. As a functional homolog of heat-shock proteins, clusterin is able to interact with a broad spectrum of partners, including the hydrophobic domains of incompletely folded or misfolded proteins [67, 68]. With such proteins, clusterin forms soluble complexes to prevent their precipitation. These complexes are thereafter targeted for degradation in the proteasome or lysosome [69, 70]. When clusterin does not exert its chaperone function, misfolded proteins can accumulate in cells, leading to apoptosis. However, 2D western blot analysis of clusterin *N*-glycosylation did not show any consistent spot pattern changes in either the ERF or blood plasma of the analyzed groups. Therefore, the change in the normalized volume of clusterin-containing spots was most probably not caused by *N*-glycosylation.

Clusterin is implicated in pathological conditions that, otherwise unrelated, are characterized by increased oxidative stress. Oxidative stress is one of the factors contributing to MDS pathogenesis, with reactive oxygen species levels increased in low-risk MDS patients [71]. Stress conditions, such as oxidative stress, destabilize the native conformation of proteins and lead to their precipitation or aggregation [68, 72]. Protein aggregates cause proteome instability by disrupting cellular homeostasis [73]. Clusterin synthesis is induced by reactive oxygen species accumulation and subsequent proteotoxic stress in order to chaperone impaired proteins and reduce cell damage [72, 74]. Under elevated oxidative stress, clusterin not only protects the cell but also might itself be the subject of oxidative modification. Such modifications may lead to the loss, change or gain of protein function. Moreover, the loss or change of clusterin function might be the reason why cells are able to evade complement attack and subsequent apoptosis, possibly by terminal complex elimination via the release of EVs [75]. In addition, EV release from impaired cells can potentially act as a healing mechanism by disposing of harmful damaged molecules [76]. However, when we looked for clusterin oxidative modifications, error-tolerant searches of both the CID and ETD data did not reveal any such modification.

**Fig 3:**
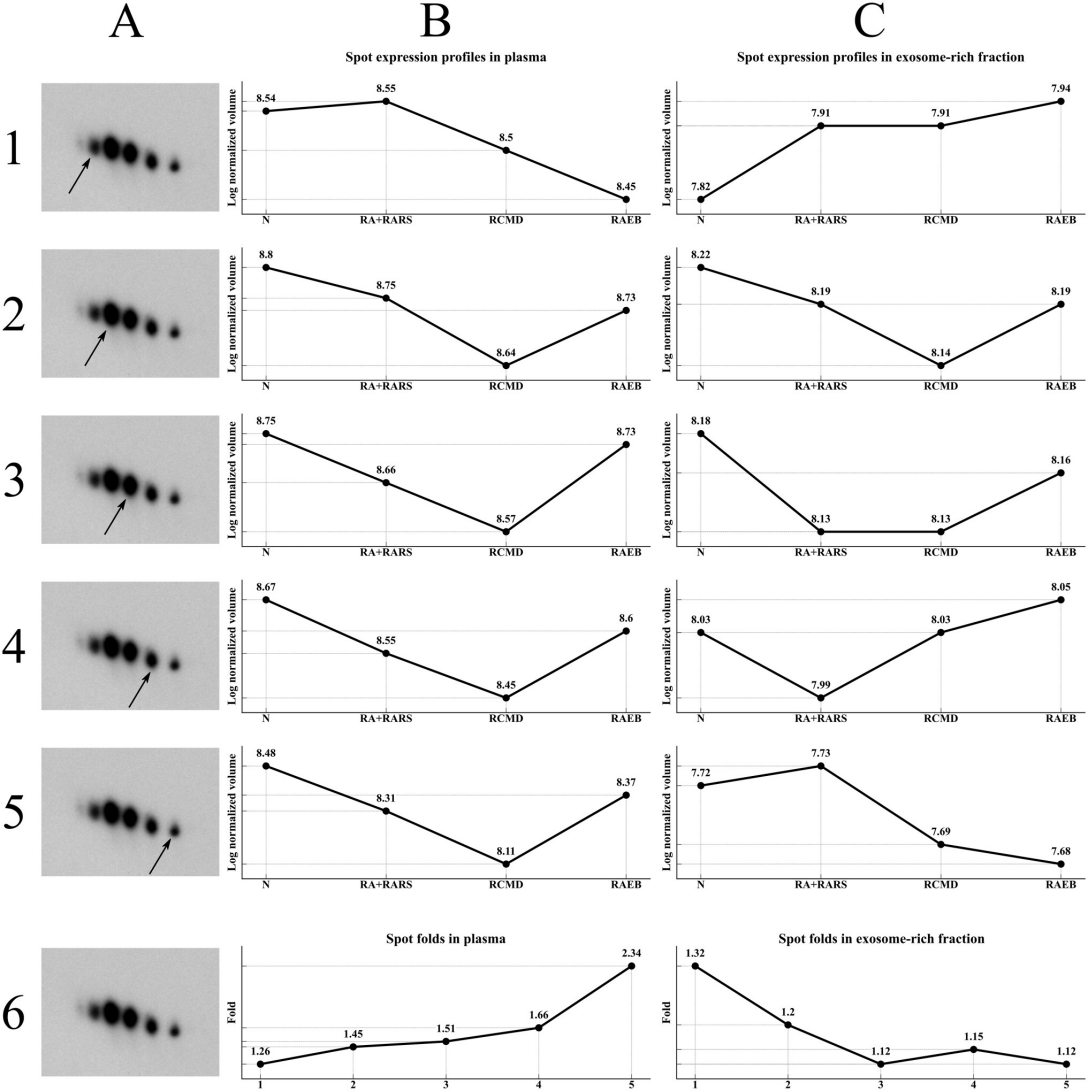
Two-dimensional western blot analysis of clusterin. Panel A represents an illustrative clusterin spot pattern. The arrow indicates the specific clusterin spot (A1–A5) with its expression profiles in plasma (B1–B5) and exosome-rich fraction (C1–C5). Fold values for each spot (A1–A5) are presented for plasma (B6) and exosome-rich fraction (C6).

Because we cannot exclude the influence of other PTMs [77–79], or mutual combinations thereof, on clusterin alteration, we used 2D western blot to analyze the spot pattern and to verify that any such change was only present in the ERF, not in the plasma. Identical changes in both the ERF and plasma would mean that we would have to exclude clusterin alterations in ERF as a potential biomarker. However, we identified one very interesting clusterin spot (Fig 3A1). In the plasma, this spot expression profile decreased with MDS severity (Fig 3B1) while in the ERF it increased with MDS severity (Fig 3C1). Whether this change was the result of a PTM or combination of PTMs, it strongly indicates the presence of a significant specific clusterin proteoform. Given the unknown nature of the modification(s) in question, it is very difficult to speculate on their possible physiological significance. This is all the more so as different modifications may have completely opposite effects.

This study has several limitations that should be mentioned. The first is the size of the patient cohort, especially for high-risk MDS patients in RAEB-1 and RAEB-2 subgroups, which is due to the fact that MDS is a rare blood disorder. Second, blood samples were centrifuged at 4,000 × g whereas the recommended value by the manufacturer of the ExoQuick kit is 3,000 × g. This could affect the composition of the isolated EVs, however the DLS data are consistent (S1 and S2 Files) with other work using a different isolation method (ultracentrifugation) [53, 54]. Third, this work does not use pure isolated exosomes but exosome-rich fraction (ERF) as explicitly mentioned in the text. However, given the DLS data, exosomes constitute the major fraction of isolated EVs.

## Conclusions

We have identified a specific difference between the plasma and ERF of MDS patients and healthy donors. In the plasma, the expression profile for this spot decreased as MDS severity increased; in the ERF, it showed the reverse trend, increasing with MDS severity. This highly interesting clusterin spot represents a significant clusterin proteoform. Because clusterin is an important participant in processes contributing to MDS pathophysiology, such as immune system dysregulation, oxidative stress and/or apoptosis, we believe that this specific form of clusterin could be a biomarker of MDS progression. However, before it can be recognized as such, it will be necessary to confirm our findings using a much larger cohort. In addition, our study opens up another avenue of research; namely, the need to identify the specific PTM, or combination of PTMs, that modifies clusterin in this way.

## Supporting information

S1 Table Database match. Match of identified proteins with exosomal protein databases.
(XLSX)

S2 Table List of spots. List of spots that differed significantly among analyzed subgroups.
(XLSX)

S1 File DLS report. DLS size distribution report by intensity.
(PDF)

S2 File DLS report. DLS size distribution report by volume.
(PDF)

S1 Raw images 2D SDS-PAGE gel image, 2D western blot of clusterin. (PDF)

## References

[1] Vardiman JW, Thiele J, Arber DA, Brunning RD, Borowitz MJ, Porwit A, et al. The 2008 revision of the World Health Organization (WHO) classification of myeloid neoplasms and acute leukemia: Rationale and important changes. Blood. 2009. pp. 937–951. doi: 10.1182/blood-2009-03-209262 19357394

[2] Brunning RD, Bennett JM, Flandrin G, Matutes E, Head D, Vardiman JW, et al. Myelodysplastic syndromes and acute myeloid leukemias. 1st ed. In: Jaffe ES, Harris NL, Stein H, Vardiman JW, editors. World Health Organization Classification of Tumours Pathology and Genetics of Tumours of Haematopoietic and Lymphoid Tissues. 1st ed. Lyon: IARC Press; 2001. pp. 61–106.

[3] Robbins PD, Morelli AE. Regulation of immune responses by extracellular vesicles. Nat Rev Immunol. 2014;14: 195–208. doi: 10.1038/nri3622 24566916 PMC4350779

[4] Utleg AG, Yi EC, Xie T, Shannon P, White JT, Goodlett DR, et al. Proteomic analysis of human prostasomes. Prostate. 2003;56: 150–161. doi: 10.1002/pros.10255 12746840

[5] Pisitkun T, Shen R-F, Knepper MA. Identification and proteomic profiling of exosomes in human urine. Proc Natl Acad Sci U S A. 2004;101: 13368–13373. doi: 10.1073/pnas.0403453101 15326289 PMC516573

[6] Caby M-P, Lankar D, Vincendeau-Scherrer C, Raposo G, Bonnerot C. Exosomal-like vesicles are present in human blood plasma. Int Immunol. 2005;17: 879–887. doi: 10.1093/intimm/dxh267 15908444

[7] Admyre C, Johansson SM, Qazi KR, Filén J-J, Lahesmaa R, Norman M, et al. Exosomes with immune modulatory features are present in human breast milk. J Immunol. 2007;179: 1969–1978. doi: 10.4049/jimmunol.179.3.1969 17641064

[8] Keller S, Rupp C, Stoeck A, Runz S, Fogel M, Lugert S, et al. CD24 is a marker of exosomes secreted into urine and amniotic fluid. Kidney Int. 2007;72: 1095–1102. doi: 10.1038/sj.ki.5002486 17700640

[9] Ogawa Y, Kanai-Azuma M, Akimoto Y, Kawakami H, Yanoshita R. Exosome-like vesicles with dipeptidyl peptidase IV in human saliva. Biol Pharm Bull. 2008;31: 1059–1062. doi: 10.1248/bpb.31.1059 18520029

[10] Choi D-S, Park JO, Jang SC, Yoon YJ, Jung JW, Choi D-Y, et al. Proteomic analysis of microvesicles derived from human colorectal cancer ascites. Proteomics. 2011;11: 2745–2751. doi: 10.1002/pmic.201100022 21630462

[11] Robert Taylor JB, Gadam SR, Perez L. 3249 Defining the Extracellular Vesicle Content of Interstitial Fluid for Blood-Free Diagnostics; Extraction Methods and Initial Characterization. J Clin Transl Sci. 2019;3: 7–7. doi: 10.1017/cts.2019.20

[12] Crescitelli R, Lässer C, Lötvall J. Isolation and characterization of extracellular vesicle subpopulations from tissues. Nat Protoc. 2021;16: 1548–1580. doi: 10.1038/s41596-020-00466-1 33495626

[13] Lewin S, Hunt S, Lambert DW. Extracellular vesicles and the extracellular matrix: A new paradigm or old news? Biochem Soc Trans. 2020;48: 2335–2345. doi: 10.1042/BST20200717 33125481

[14] Théry C, Zitvogel L, Amigorena S. Exosomes: Composition, biogenesis and function. Nat Rev Immunol. 2002;2: 569–579. doi: 10.1038/nri855 12154376

[15] Théry C, Ostrowski M, Segura E. Membrane vesicles as conveyors of immune responses. Nat Rev Immunol. 2009;9: 581–593. doi: 10.1038/nri2567 19498381

[16] Yang C, Robbins PD. Immunosuppressive exosomes: A new approach for treating arthritis. Int J Rheumatol. 2012;2012. doi: 10.1155/2012/573528 22548070 PMC3324137

[17] El Andaloussi S, Mäger I, Breakefield XO, Wood MJA. Extracellular vesicles: Biology and emerging therapeutic opportunities. Nat Rev Drug Discov. 2013;12: 347–357. doi: 10.1038/nrd3978 23584393

[18] Kumar B, Garcia M, Murakami JL, Chen C-C. Exosome-mediated microenvironment dysregulation in leukemia. Biochim Biophys Acta—Mol Cell Res. 2016;1863: 464–470. doi: 10.1016/j.bbamcr.2015.09.017 26384870

[19] Maas SLN, Breakefield XO, Weaver AM. Extracellular Vesicles: Unique Intercellular Delivery Vehicles. Trends Cell Biol. 2017;27: 172–188. doi: 10.1016/j.tcb.2016.11.003 27979573 PMC5318253

[20] Yoon YJ, Kim OY, Gho YS. Extracellular vesicles as emerging intercellular communicasomes. BMB Rep. 2014;47: 531–539. doi: 10.5483/bmbrep.2014.47.10.164 25104400 PMC4261509

[21] Raposo G, Nijman HW, Stoorvogel W, Leijendekker R, Harding CV, Melief CJM, et al. B lymphocytes secrete antigen-presenting vesicles. J Exp Med. 1996;183: 1161–1172. doi: 10.1084/jem.183.3.1161 8642258 PMC2192324

[22] Ratajczak J, Miekus K, Kucia M, Zhang J, Reca R, Dvorak P, et al. Embryonic stem cell-derived microvesicles reprogram hematopoietic progenitors: Evidence for horizontal transfer of mRNA and protein delivery. Leukemia. 2006;20: 847–856. doi: 10.1038/sj.leu.2404132 16453000

[23] Gatti S, Bruno S, Deregibus MC, Sordi A, Cantaluppi V, Tetta C, et al. Microvesicles derived from human adult mesenchymal stem cells protect against ischaemia-reperfusion-induced acute and chronic kidney injury. Nephrol Dial Transplant. 2011;26: 1474–1483. doi: 10.1093/ndt/gfr015 21324974

[24] Del Conde I, Shrimpton CN, Thiagarajan P, López JA. Tissue-factor-bearing microvesicles arise from lipid rafts and fuse with activated platelets to initiate coagulation. Blood. 2005;106: 1604–1611. doi: 10.1182/blood-2004-03-1095 15741221

[25] Aharon A, Rebibo-Sabbah A, Tzoran I, Levin C. Extracellular vesicles in hematological disorders. Rambam Maimonides Med J. 2014;5: e0032. doi: 10.5041/RMMJ.10166 25386348 PMC4222421

[26] Ghosh AK, Secreto CR, Knox TR, Ding W, Mukhopadhyay D, Kay NE. Circulating microvesicles in B-cell chronic lymphocytic leukemia can stimulate marrow stromal cells: Implications for disease progression. Blood. 2010;115: 1755–1764. doi: 10.1182/blood-2009-09-242719 20018914 PMC2832808

[27] Corrado C, Raimondo S, Saieva L, Flugy AM, De Leo G, Alessandro R. Exosome-mediated crosstalk between chronic myelogenous leukemia cells and human bone marrow stromal cells triggers an Interleukin 8-dependent survival of leukemia cells. Cancer Lett. 2014;348: 71–76. doi: 10.1016/j.canlet.2014.03.009 24657661

[28] Milane L, Singh A, Mattheolabakis G, Suresh M, Amiji MM. Exosome mediated communication within the tumor microenvironment. J Control Release. 2015;219: 278–294. doi: 10.1016/j.jconrel.2015.06.029 26143224

[29] Choi D-S, Kim D-K, Kim Y-K, Gho YS. Proteomics, transcriptomics and lipidomics of exosomes and ectosomes. Proteomics. 2013;13: 1554–1571. doi: 10.1002/pmic.201200329 23401200

[30] Looze C, Yui D, Leung L, Ingham M, Kaler M, Yao X, et al. Proteomic profiling of human plasma exosomes identifies PPARγ as an exosome-associated protein. Biochem Biophys Res Commun. 2009;378: 433–438. doi: 10.1016/j.bbrc.2008.11.050 19028452 PMC2633355

[31] Ramacciotti E, Hawley AE, Wrobleski SK, Myers DD Jr., Strahler JR, Andrews PC, et al. Proteomics of microparticles after deep venous thrombosis. Thromb Res. 2010;125. doi: 10.1016/j.thromres.2010.01.019 20156641 PMC2929804

[32] Raimondo F, Morosi L, Chinello C, Magni F, Pitto M. Advances in membranous vesicle and exosome proteomics improving biological understanding and biomarker discovery. Proteomics. 2011;11: 709–720. doi: 10.1002/pmic.201000422 21241021

[33] Roccaro AM, Sacco A, Maiso P, Azab AK, Tai Y-T, Reagan M, et al. BM mesenchymal stromal cell-derived exosomes facilitate multiple myeloma progression. J Clin Invest. 2013;123: 1542–1555. doi: 10.1172/JCI66517 23454749 PMC3613927

[34] Yao Y, Wei W, Sun J, Chen L, Deng X, Ma L, et al. Proteomic analysis of exosomes derived from human lymphoma cells. Eur J Med Res. 2015;20. doi: 10.1186/s40001-014-0082-4 25631545 PMC4329659

[35] Paggetti J, Haderk F, Seiffert M, Janji B, Distler U, Ammerlaan W, et al. Exosomes released by chronic lymphocytic leukemia cells induce the transition of stromal cells into cancer-associated fibroblasts. Blood. 2015;126: 1106–1117. doi: 10.1182/blood-2014-12-618025 26100252 PMC4560344

[36] Kumar B, Zhang L, Miao Y, Wuenschell G, Lin A, Pullarkat V, et al. Proteomics Profiling of Leukemia Derived Exosomes: A Potential Role in Leukemic Transformation. Blood. 2015;100: 2292–2302. doi: 10.1182/blood.V126.23.3857.3857

[37] Vardiman JW, Harris NL, Brunning RD. The World Health Organization (WHO) classification of the myeloid neoplasms. Blood. 2002;100: 2292–2302. doi: 10.1182/blood-2002-04-1199 12239137

[38] Hrustincova A, Krejcik Z, Kundrat D, Szikszai K, Belickova M, Pecherkova P, et al. Circulating Small Noncoding RNAs Have Specific Expression Patterns in Plasma and Extracellular Vesicles in Myelodysplastic Syndromes and Are Predictive of Patient Outcome. Cells. 2020;9. doi: 10.3390/cells9040794 32224889 PMC7226126

[39] Májek P, Reicheltová Z, Štikarová J, Suttnar J, Sobotková A, Dyr JE. Proteome changes in platelets activated by arachidonic acid, collagen, and thrombin. Proteome Sci. 2010;8. doi: 10.1186/1477-5956-8-56 21073729 PMC2996359

[40] Chevallet M, Luche S, Diemer H, Strub J-M, Van Dorsselaer A, Rabilloud T. Sweet silver: A formaldehyde-free silver staining using aldoses as developing agents, with enhanced compatibility with mass spectrometry. Proteomics. 2008;8: 4853–4861. doi: 10.1002/pmic.200800321 19003863

[41] Breuza L, Poux S, Estreicher A, Famiglietti ML, Magrane M, Tognolli M, et al. The UniProtKB guide to the human proteome. Database. 2016;2016. doi: 10.1093/database/bav120 26896845 PMC4761109

[42] Mathivanan S, Simpson RJ. ExoCarta: A compendium of exosomal proteins and RNA. Proteomics. 2009;9: 4997–5000. doi: 10.1002/pmic.200900351 19810033

[43] Mathivanan S, Fahner CJ, Reid GE, Simpson RJ. ExoCarta 2012: Database of exosomal proteins, RNA and lipids. Nucleic Acids Res. 2012;40. doi: 10.1093/nar/gkr828 21989406 PMC3245025

[44] Simpson RJ, Kalra H, Mathivanan S. Exocarta as a resource for exosomal research. J Extracell Vesicles. 2012;1. doi: 10.3402/jev.v1i0.18374 24009883 PMC3760644

[45] Keerthikumar S, Chisanga D, Ariyaratne D, Al Saffar H, Anand S, Zhao K, et al. ExoCarta: A Web-Based Compendium of Exosomal Cargo. J Mol Biol. 2016;428: 688–692. doi: 10.1016/j.jmb.2015.09.019 26434508 PMC4783248

[46] Binns D, Dimmer E, Huntley R, Barrell D, O’Donovan C, Apweiler R. QuickGO: A web-based tool for Gene Ontology searching. Bioinformatics. 2009;25: 3045–3046. doi: 10.1093/bioinformatics/btp536 19744993 PMC2773257

[47] Zielinska DF, Gnad F, Wiśniewski JR, Mann M. Precision mapping of an in vivo N-glycoproteome reveals rigid topological and sequence constraints. Cell. 2010;141: 897–907. doi: 10.1016/j.cell.2010.04.012 20510933

[48] Boyiadzis M, Whiteside TL. Plasma-derived exosomes in acute myeloid leukemia for detection of minimal residual disease: are we ready? Expert Rev Mol Diagn. 2016;16: 623–629. doi: 10.1080/14737159.2016.1174578 27043038 PMC5400097

[49] Pandey KB, Rizvi SI. Biomarkers of oxidative stress in red blood cells. Biomed Pap. 2011;155: 131–136. doi: 10.5507/bp.2011.027 21804621

[50] Westerman M, Porter JB. Red blood cell-derived microparticles: An overview. Blood Cells, Mol Dis. 2016;59: 134–139. doi: 10.1016/j.bcmd.2016.04.003 27282583

[51] Halim ATA, Ariffin NAFM, Azlan M. Review: the Multiple Roles of Monocytic Microparticles. Inflammation. 2016;39: 1277–1284. doi: 10.1007/s10753-016-0381-8 27216803

[52] Panteleev MA, Abaeva AA, Balandina AN, Belyaev AV, Nechipurenko DY, Obydennyi SI, et al. Extracellular vesicles of blood plasma: content, origin, and properties. Biochem Suppl Ser A Membr Cell Biol. 2017;11: 187–192. doi: 10.1134/S1990747817030060

[53] Abello J, Nguyen TDT, Marasini R, Aryal S, Weiss ML. Biodistribution of gadolinium- and near infrared-labeled human umbilical cord mesenchymal stromal cell-derived exosomes in tumor bearing mice. Theranostics. 2019;9: 2325–2345. doi: 10.7150/thno.30030 31149047 PMC6531310

[54] Giassafaki L-PN, Siqueira S, Panteris E, Psatha K, Chatzopoulou F, Aivaliotis M, et al. Towards analyzing the potential of exosomes to deliver microRNA therapeutics. J Cell Physiol. 2021;236: 1529–1544. doi: 10.1002/jcp.29991 32749687

[55] McLellan A.D. Exosome release by primary B cells. Crit Rev Immunol. 2009;29: 203–217. doi: 10.1615/critrevimmunol.v29.i3.20 19538135

[56] Villarroya-Beltri C, Baixauli F, Gutiérrez-Vázquez C, Sánchez-Madrid F, Mittelbrunn M. Sorting it out: Regulation of exosome loading. Semin Cancer Biol. 2014;28: 3–13. doi: 10.1016/j.semcancer.2014.04.009 24769058 PMC4640178

[57] Sódar BW, Kittel Á, Pálóczi K, Vukman KV, Osteikoetxea X, Szabó-Taylor K, et al. Low-density lipoprotein mimics blood plasma-derived exosomes and microvesicles during isolation and detection. Sci Rep. 2016;6. doi: 10.1038/srep24316 27087061 PMC4834552

[58] Karimi N, Cvjetkovic A, Jang SC, Crescitelli R, Hosseinpour Feizi MA, Nieuwland R, et al. Detailed analysis of the plasma extracellular vesicle proteome after separation from lipoproteins. Cell Mol Life Sci. 2018;75: 2873–2886. doi: 10.1007/s00018-018-2773-4 29441425 PMC6021463

[59] Onódi Z, Pelyhe C, Nagy CT, Brenner GB, Almási L, Kittel Á, et al. Isolation of high-purity extracellular vesicles by the combination of iodixanol density gradient ultracentrifugation and bind-elute chromatography from blood plasma. Front Physiol. 2018;9. doi: 10.3389/fphys.2018.01479 30405435 PMC6206048

[60] Record M, Carayon K, Poirot M, Silvente-Poirot S. Exosomes as new vesicular lipid transporters involved in cell-cell communication and various pathophysiologies. Biochim Biophys Acta—Mol Cell Biol Lipids. 2014;1841: 108–120. doi: 10.1016/j.bbalip.2013.10.004 24140720

[61] Zipfel PF, Skerka C. Complement regulators and inhibitory proteins. Nat Rev Immunol. 2009;9: 729–740. doi: 10.1038/nri2620 19730437

[62] Tschopp J, Chonn A, Hertig S, French LE. Clusterin, the human apolipoprotein and complement inhibitor, binds to complement C7, C8β, and the b domain of C9. J Immunol. 1993;151: 2159–2165. 8345200

[63] Kurosu T, Chaichana P, Yamate M, Anantapreecha S, Ikuta K. Secreted complement regulatory protein clusterin interacts with dengue virus nonstructural protein 1. Biochem Biophys Res Commun. 2007;362: 1051–1056. doi: 10.1016/j.bbrc.2007.08.137 17825259

[64] Rodríguez-Piñeiro AM, Páez de la Cadena M, López-Saco Á, Rodríguez-Berrocal FJ. Differential expression of serum clusterin isoforms in colorectal cancer. Mol Cell Proteomics. 2006;5: 1647–1657. doi: 10.1074/mcp.M600143-MCP200 16854844

[65] Rizzi F, Caccamo AE, Belloni L, Bettuzzi S. Clusterin is a short half-life, poly-ubiquitinated protein, which controls the fate of prostate cancer cells. J Cell Physiol. 2009;219: 314–323. doi: 10.1002/jcp.21671 19137541

[66] Matukumalli SR, Tangirala R, Rao CM. Clusterin: Full-length protein and one of its chains show opposing effects on cellular lipid accumulation. Sci Rep. 2017;7. doi: 10.1038/srep41235 28120874 PMC5264606

[67] Wilson MR, Easterbrook-Smith SB. Clusterin is a secreted mammalian chaperone. Trends Biochem Sci. 2000;25: 95–98. doi: 10.1016/s0968-0004(99)01534-0 10694874

[68] Poon S, Treweek TM, Wilson MR, Easterbrook-Smith SB, Carver JA. Clusterin is an extracellular chaperone that specifically interacts with slowly aggregating proteins on their off-folding pathway. FEBS Lett. 2002;513: 259–266. doi: 10.1016/s0014-5793(02)02326-8 11904161

[69] Soti C, Pál C, Papp B, Csermely P. Molecular chaperones as regulatory elements of cellular networks. Curr Opin Cell Biol. 2005;17: 210–215. doi: 10.1016/j.ceb.2005.02.012 15780599

[70] Wilson MR, Zoubeidi A. Clusterin as a therapeutic target. Expert Opin Ther Targets. 2017;21: 201–213. doi: 10.1080/14728222.2017.1267142 27978767

[71] Gonçalves AC, Cortesão E, Oliveiros B, Alves V, Espadana AI, Rito L, et al. Oxidative stress and mitochondrial dysfunction play a role in myelodysplastic syndrome development, diagnosis, and prognosis: A pilot study. Free Radic Res. 2015;49: 1081–1094. doi: 10.3109/10715762.2015.1035268 25968944

[72] Kim JH, Kim JH, Jun HO, Yu YS, Min BH, Park KH, et al. Protective effect of clusterin from oxidative stress-induced apoptosis in human retinal pigment epithelial cells. Investig Ophthalmol Vis Sci. 2010;51: 561–566. doi: 10.1167/iovs.09-3774 19710412

[73] Trougakos IP. The molecular chaperone apolipoprotein J/Clusterin as a sensor of oxidative stress: Implications in therapeutic approaches—A mini-review. Gerontology. 2013;59: 514–523. doi: 10.1159/000351207 23689375

[74] Trougakos IP, Gonos ES. Oxidative stress in malignant progression: The role of clusterin, a sensitive cellular biosensor of free radicals. Advances in Cancer Research. 2009. doi: 10.1016/S0065-230X(09)04009-3 19878777

[75] Pilzer D, Gasser O, Moskovich O, Schifferli JA, Fishelson Z. Emission of membrane vesicles: Roles in complement resistance, immunity and cancer. Springer Semin Immunopathol. 2005;27: 375–387. doi: 10.1007/s00281-005-0004-1 16189651

[76] Abid Hussein MN, Böing AN, Sturk A, Hau CM, Nieuwland R. Inhibition of microparticle release triggers endothelial cell apoptosis and detachment. Thromb Haemost. 2007;98: 1096–1107. doi: 10.1160/th05-04-0231 18000616

[77] Lemansky P, Brix K, Herzog V. Subcellular distribution, secretion, and posttranslational modifications of clusterin in thyrocytes. Exp Cell Res. 1999;251: 147–155. doi: 10.1006/excr.1999.4555 10438580

[78] O’Sullivan J, Whyte L, Drake J, Tenniswood M. Alterations in the post-translational modification and intracellular trafficking of clusterin in MCF-7 cells during apoptosis. Cell Death Differ. 2003;10: 914–927. doi: 10.1038/sj.cdd.4401254 12867999

[79] Bian Y, Song C, Cheng K, Dong M, Wang F, Huang J, et al. An enzyme assisted RP-RPLC approach for in-depth analysis of human liver phosphoproteome. J Proteomics. 2014;96: 253–262. doi: 10.1016/j.jprot.2013.11.014 24275569

