# Supplementary material for: Proteome changes of plasma-derived extracellular vesicles in patients with myelodysplastic syndrome
Source: PLoS One. 2022 Jan 10;17(1):e0262484. doi: 10.1371/journal.pone.0262484 (PMC8746746; doi:10.1371/journal.pone.0262484)

Size Distribution Report by Intensity

v2.2

Malvern

Sample Details

Sample Name: Exosomes 4x no filtrartion 10x dil

SOP Name: mansettings.nano

General Notes: PBS 7.4

**File Name:**	Exosomes 07.09.15	**Dispersant Name:**	Water
**Record Number:**	3	**Dispersant RI:**	1.330
**Material RI:**	1.59	**Viscosity (cP):**	0.8872
**Material Absorbtion:**	0.010	**Measurement Date and Time:**	Monday, September 07, 20...

System

**Temperature (°C):**	25.1	**Duration Used (s):**	30
**Count Rate (kcps):**	400.9	**Measurement Position (mm):**	1.25
**Cell Description:**	Low volume disposable sizin...	**Attenuator:**	7

Results

			Size (d.n...	% Intensity:	St Dev (d.n...
**Z-Average (d.nm):**	141.5	**Peak 1:**	177.6	73.1	69.60
**PdI:**	0.265	**Peak 2:**	39.85	26.9	13.36
**Intercept:**	0.935	**Peak 3:**	0.000	0.0	0.000

Result quality **Refer to quality report**

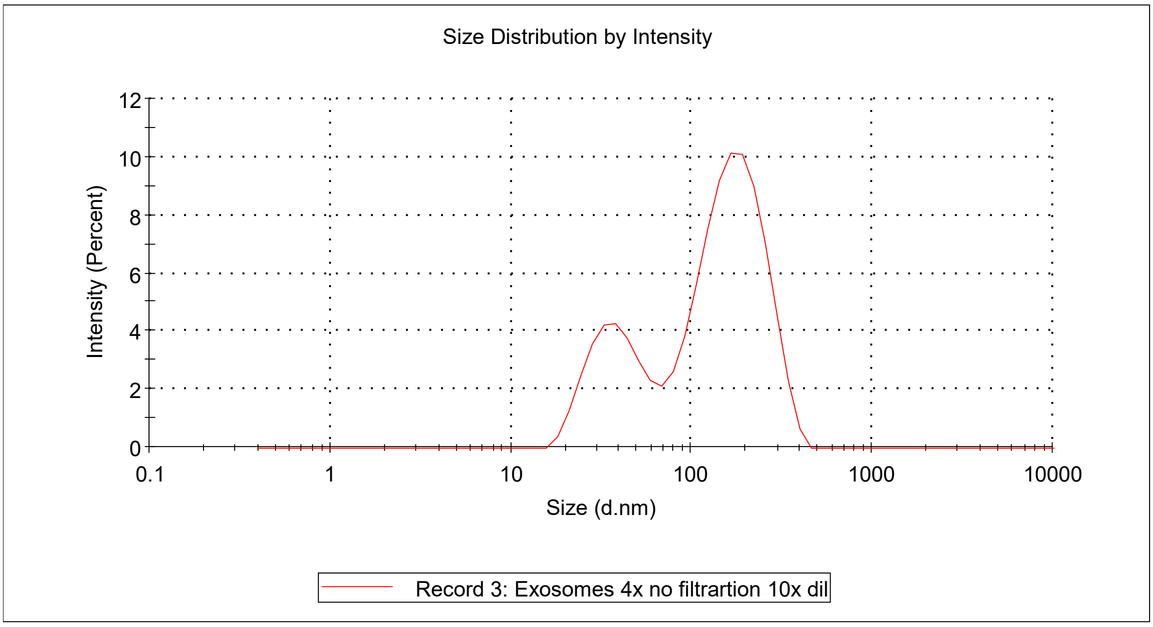

Supplement: S1 File — DLS size distribution report by intensity. (PDF) [file pone.0262484.s003.pdf]
