# Supplementary material for: Proteome changes of plasma-derived extracellular vesicles in patients with myelodysplastic syndrome
Source: PLoS One. 2022 Jan 10;17(1):e0262484. doi: 10.1371/journal.pone.0262484 (PMC8746746; doi:10.1371/journal.pone.0262484)

Size Distribution Report by Volume

v2.2

Sample Details

**Sample Name:**	Exosomes 4x no filtrartion 10x dil
**SOP Name:**	mansettings.nano
**General Notes:**	PBS 7.4

**File Name:**	Exosomes 07.09.15	**Dispersant Name:**	Water
**Record Number:**	3	**Dispersant RI:**	1.330
**Material RI:**	1.59	**Viscosity (cP):**	0.8872
**Material Absorbtion:**	0.010	**Measurement Date and Time:**	Monday, September 07, 2...

System

**Temperature (°C):**	25.1	**Duration Used (s):**	30
**Count Rate (kcps):**	400.9	**Measurement Position (mm):**	1.25
**Cell Description:**	Low volume disposable sizi...	**Attenuator:**	7

Results

			**Size (d.nm):**	**% Volume:**	**St Dev (d.nm):**
**Z-Average (d.nm):**	141.5	**Peak 1:**	30.42	92.8	12.00
**PdI:**	0.265	**Peak 2:**	115.5	3.0	18.78
**Intercept:**	0.935	**Peak 3:**	211.4	4.2	62.97
**Result quality**	**Refer to quality report**				

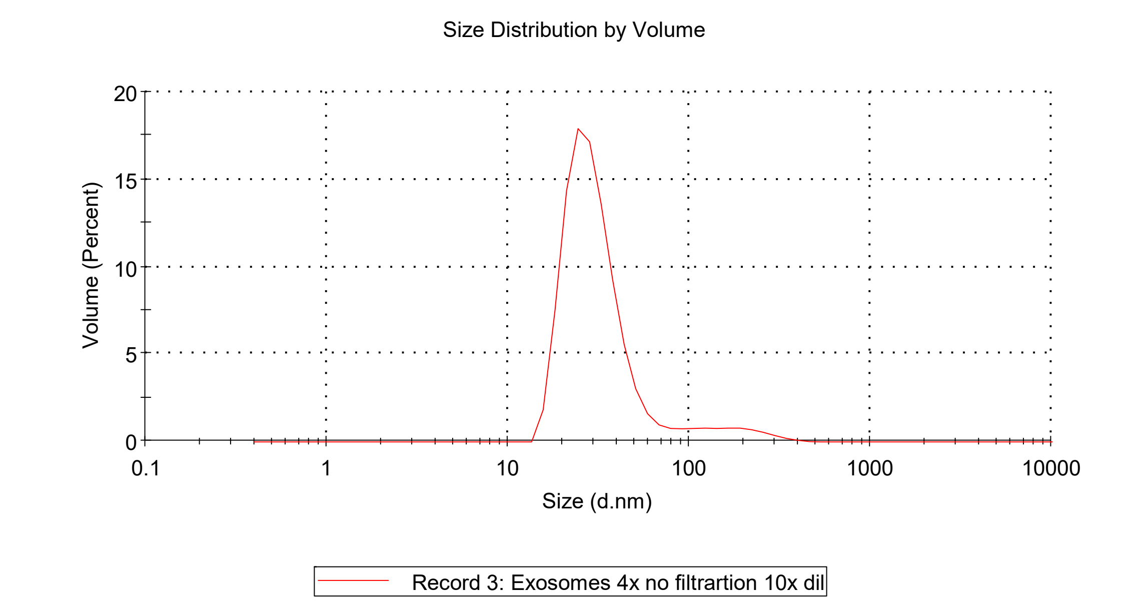

Supplement: S2 File — DLS size distribution report by volume. (PDF) [file pone.0262484.s004.pdf]
