# Supplementary material for: Proteome changes of plasma-derived extracellular vesicles in patients with myelodysplastic syndrome
Source: PLoS One. 2022 Jan 10;17(1):e0262484. doi: 10.1371/journal.pone.0262484 (PMC8746746; doi:10.1371/journal.pone.0262484)
Supplement: S1 Raw images — (PDF) [file pone.0262484.s005.pdf]

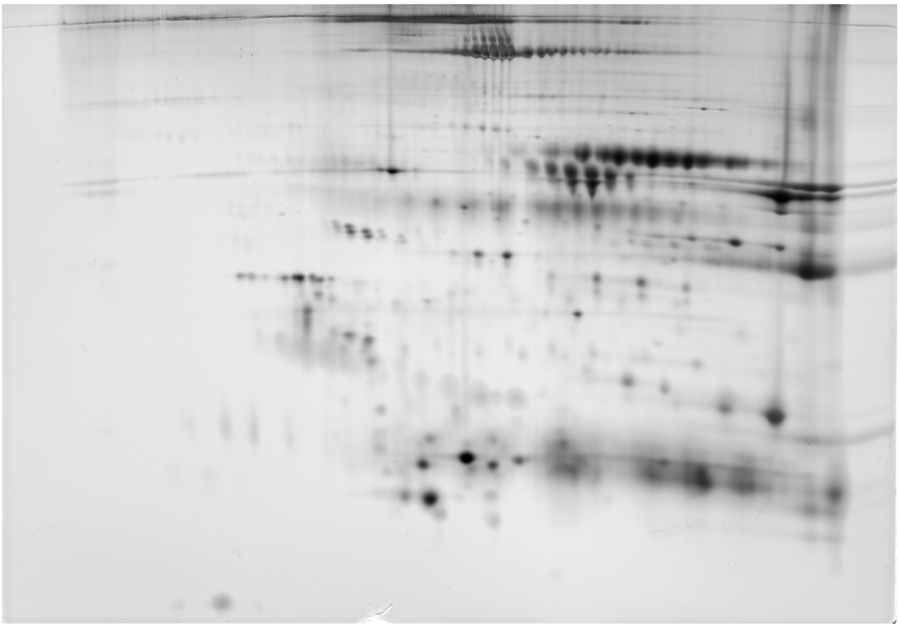

raw image of Fig 2

Sample: MDS
Gel: pI 4-7, 10%
Staining: silver
Scanned: 1200 dpi,
16-bit grayscale

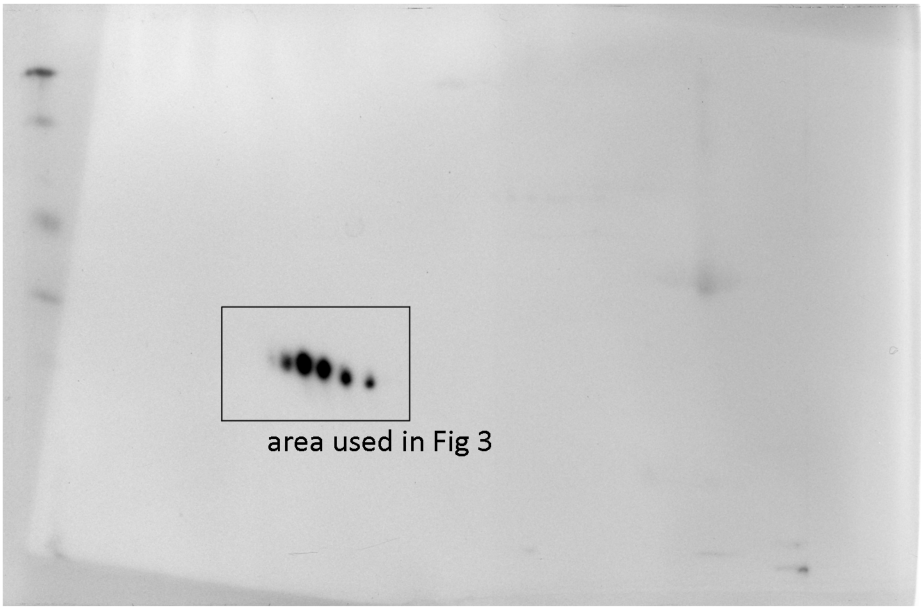

raw image of Fig 3

Sample: MDS
Gel: pI 4-7, 10%
Membrane: PVDF
Film: CL-XPosure
Exposition: 10 min
Scanned: 1200 dpi,
16-bit grayscale
